# Effects of a 12-week mini-basketball exercise intervention on executive function in children with attention-deficit/hyperactivity disorder: a randomized controlled trial

**DOI:** 10.3389/fpsyg.2026.1840290

**Published:** 2026-05-21

**Authors:** Chengcheng Cai, Ruinan Liu, Yingbo Zhu, Zehui Wen

**Affiliations:** 1 Tennis Professional College, Wuhan City Polytechnic, Wuhan, China; 2 School of Athletic Performance, Shanghai University of Sport, Shanghai, China; 3 School of Physical Education and Sport, Henan University, Kaifeng, China

**Keywords:** ADHD, children, executive function, Mini-basketball, randomized controlled trial

## Abstract

**Background:**

Executive function (EF) deficits are a core cognitive impairment in children with ADHD. While physical exercise has been shown to benefit EF, the effects of cognitively engaging, sport-based interventions remain insufficiently understood. This study examined the effects of a 12-week mini-basketball intervention on EF in children with ADHD using a randomized controlled trial design.

**Methods:**

Thirty-five children aged 6–11 years meeting DSM-5 criteria for ADHD were randomly assigned to an exercise group (EG, *n* = 18) or a control group (CG, *n* = 17). The EG participated in a 12-week moderate-intensity mini-basketball program (3 sessions/week, 45 min/session), whereas the CG maintained usual activities. Inhibitory control (IC), working memory (WM), and global EF were assessed at baseline and post-intervention using the Childhood Executive Functioning Inventory (CHEXI). Between-group differences were analyzed using ANCOVA controlling for baseline values.

**Results:**

Compared with the CG, the EG demonstrated significantly greater improvements in IC (*F* = 15.15, *p* < 0.001, partial η^2^ = 0.35), WM (*F* = 30.55, *p* < 0.001, partial η^2^ = 0.52), and global EF (*F* = 40.68, *p* < 0.001, partial η^2^ = 0.59), all indicating large effect sizes. Within-group analyses showed significant improvements across all EF domains in the EG (all *p* < 0.05), with no significant changes observed in the CG.

**Conclusion:**

A 12-week mini-basketball intervention significantly enhanced multiple domains of EF in children with ADHD. These findings highlight the potential of cognitively engaging, sport-based exercise as an effective and feasible non-pharmacological strategy for improving executive function in this population. However, the relatively small sample size and reliance on parent-reported measures should be considered when interpreting the results.

## Highlights

- Conducted a 12-week mini-basketball intervention targeting children with ADHD to improve their executive function, including inhibitory control and working memory.
- Demonstrated that regular participation in the program significantly enhanced key cognitive skills compared to a control group receiving standard care.
- One of the first randomized controlled trials to evaluate the effects of a structured mini-basketball program on cognitive function, offering a feasible non-pharmacological intervention for child neurodevelopment.

## 1 Introduction

Attention-deficit/hyperactivity disorder (ADHD) is among the most prevalent neurodevelopmental disorders in childhood, characterized by developmentally inappropriate levels of inattention, hyperactivity, and impulsivity (Goldman et al., 1998; American Psychiatric Association, 2022). Recent high-level evidence, including systematic reviews and meta-analyses, indicates that ADHD affects approximately 5–8% of children and adolescents worldwide, with a prevalence in boys about twice that observed in girls (Ayano et al., 2023; Salari et al., 2023). Moreover, in a substantial proportion of individuals, ADHD symptoms persist into adolescence and even adulthood rather than remitting naturally with age. For example, Van Meter et al. (2024), in a longitudinal study, found that approximately 60–70% of children diagnosed with ADHD continue to exhibit symptoms through adolescence and young adulthood. These enduring functional impairments significantly impact academic performance, interpersonal relationships, and occupational functioning (Faraone et al., 2006; Barkley et al., 2002), making ADHD a pressing global public health concern.

Although the clinical diagnosis of ADHD is primarily based on behavioral criteria, a growing body of research suggests that these observable behaviors reflect underlying abnormalities in cognitive control processes (Kieling et al., 2008). Among various theoretical models proposed to explain the cognitive mechanisms of ADHD, deficits in executive function have been identified as one of the most consistently supported core cognitive features (Barkley, 1997; Willcutt et al., 2005). Recent meta-analytic and systematic review evidence further supports this view, indicating that children with ADHD exhibit significant and widespread impairments in executive function across multiple domains (Ceruti et al., 2024; Sadozai et al., 2024). Executive function (EF) refers to a set of higher-order cognitive processes that enable individuals to regulate cognition and behavior in a goal-directed manner (Diamond, 2013). In line with influential theoretical frameworks, such as the unity and diversity model proposed by Miyake et al. (2000), EF can be understood as comprising several partially independent but interrelated components, including inhibitory control, working memory, and cognitive flexibility, which jointly support adaptive and goal-directed behavior. Previous studies have consistently shown that children and adolescents with ADHD exhibit impairments in suppressing irrelevant responses and maintaining/updating working memory representations (Willcutt et al., 2005; Kofler et al., 2024; Biederman et al., 2004), which are closely associated with attentional regulation, self-control, and overall functional outcomes. Consequently, executive dysfunction is widely regarded as a critical neurocognitive basis for understanding the behavioral phenotype of ADHD and has become a key target in intervention research.

Currently, pharmacological treatment and behavioral or psychological interventions remain the primary approaches for alleviating core symptoms and functional impairments in children with ADHD. While pharmacological interventions can effectively reduce certain symptoms in the short term, concerns remain regarding long-term efficacy, treatment adherence, and sustainability (Faraone et al., 2021). Behavioral and psychological interventions have also demonstrated positive effects on functional outcomes; however, their implementation typically requires long-term, intensive professional support, which may limit scalability and feasibility in real-world settings (Sonuga-Barke et al., 2013). Accordingly, identifying safe, feasible, and sustainable non-pharmacological strategies has become an important direction in contemporary ADHD intervention research.

Physical exercise has emerged as a promising non-pharmacological intervention due to its favorable safety profile and high acceptability, with increasing attention directed toward its potential role in promoting EF development in children with ADHD. Existing evidence suggests that both acute and long-term exercise interventions can improve EF performance in this population (Vysniauske et al., 2020; Pontifex et al., 2013). For example, regular moderate-intensity aerobic exercise has been shown to enhance overall EF (Chang et al., 2012), while interventions involving specific sports such as swimming, judo, and football have reported positive effects on inhibitory control and working memory (Chang et al., 2014; Ludyga et al., 2022; Song Y. L. et al., 2022). Nevertheless, the magnitude and consistency of these effects vary across studies, suggesting that the impact of exercise on EF is likely influenced by multiple factors, including exercise modality, task structure, and the level of cognitive engagement during activity (Best, 2010; Mao et al., 2024). Recent research has further proposed that physical activities characterized by higher cognitive engagement may confer greater benefits for EF development than simple, repetitive forms of exercise (Mao et al., 2024; Song W. et al., 2022). Mini-basketball, a form of basketball specifically designed for children, features clear rules, rapidly changing game contexts, and an emphasis on teamwork and real-time decision-making, aligning well with cognitively engaging physical activities. While ensuring safety and enjoyment, mini-basketball requires children to continuously monitor dynamic environmental information, inhibit irrelevant responses, and flexibly update action plans in response to game demands (Best, 2010; Contreras-Osorio et al., 2021; Xu et al., 2022; Li et al., 2024), thereby placing sustained demands on attentional resources, inhibitory control, and working memory. These characteristics suggest that mini-basketball may be particularly well-suited to promoting overall executive function and its core components.

However, empirical evidence specifically examining mini-basketball-based interventions in children with ADHD remains limited. To date, only a small number of randomized controlled trials have reported preliminary evidence suggesting potential improvements in executive function in children with ADHD following basketball-related training (Eskandarnejad et al., 2015). These studies primarily focus on general forms of basketball and may not be directly generalizable to developmentally adapted mini-basketball programs designed for children. In addition, they are constrained by relatively small sample sizes and limited methodological diversity. Consequently, the mechanisms through which cognitively engaging sports such as mini-basketball influence EF in children with ADHD remain largely theoretical and have not been directly established. These limitations highlight the need for further well-designed studies to clarify the underlying pathways. The present study adopts a randomized controlled trial design to evaluate the effects of a 12-week mini-basketball intervention on EF in children with ADHD. It is hypothesized that, compared with a control group maintaining usual daily activities, children with ADHD who participate in the intervention will demonstrate greater improvements in inhibitory control, working memory, and overall EF. This study aims to address the current lack of evidence regarding cognitively engaging sport-based interventions in the non-pharmacological treatment of ADHD and to further contribute to the evidence base for cognitively engaging sport-based interventions in ADHD.

## 2 Methods

### 2.1 Study design

This study was conducted and reported in accordance with the Consolidated Standards of Reporting Trials (CONSORT) guidelines for randomized, non-pharmacological intervention studies. A parallel-group randomized controlled trial (RCT) with a pretest–posttest design was employed to examine the effects of a 12-week mini-basketball exercise intervention on EF in children with ADHD. Participants were randomly allocated in a 1:1 ratio to either an exercise group (EG), which received the structured mini-basketball exercise program, or a wait-list control group (CG), which continued their usual daily activities without participation in any additional structured exercise or behavioral interventions during the study period. The intervention lasted 12 weeks, and outcome assessments were conducted at baseline (1 week prior to the intervention) and immediately after the completion of the intervention.

### 2.2 Participants

Children with ADHD were recruited between May and June 2024 with the assistance of the Department of Behavioral Psychology at Kaifeng Children’s Hospital, Henan Province, China. All participants had received a confirmed clinical diagnosis of ADHD within the previous year, and the hospital facilitated participant identification and referral based on existing clinical records. An *a priori* sample size estimation was conducted using G*Power (version 3.1.9). Based on a medium effect size (*f* = 0.40) derived from previous studies, a significance level of *α* = 0.05, and statistical power (1 − *β*) = 0.80 for a two-tailed test, the required sample size was estimated to be 44 participants. Allowing for an anticipated dropout rate of approximately 20%, the target sample size was increased to at least 53 participants. However, due to practical constraints in recruitment, the final sample size did not reach the target, which may have reduced statistical power and should be considered when interpreting the findings. Therefore, the findings should be interpreted as preliminary.

Participants were screened according to predefined inclusion and exclusion criteria. Inclusion criteria were: (1) age 6–12 years; (2) diagnosis of ADHD according to DSM-5 criteria by a qualified psychiatrist; (3) intelligence quotient (IQ) ≥ 70; (4) ability to safely participate in moderate-intensity physical activity; (5) not currently receiving pharmacological treatment or having discontinued medication for at least 3 months; and (6) no participation in structured exercise or behavioral interventions within the previous 3 months. Exclusion criteria included: (1) comorbid neurological or psychiatric disorders; (2) severe sensory impairments; (3) physical conditions contraindicating exercise; (4) obesity exceeding age-specific criteria; and (5) high habitual physical activity levels (PARS-3 score > 43).

**Figure 1:**
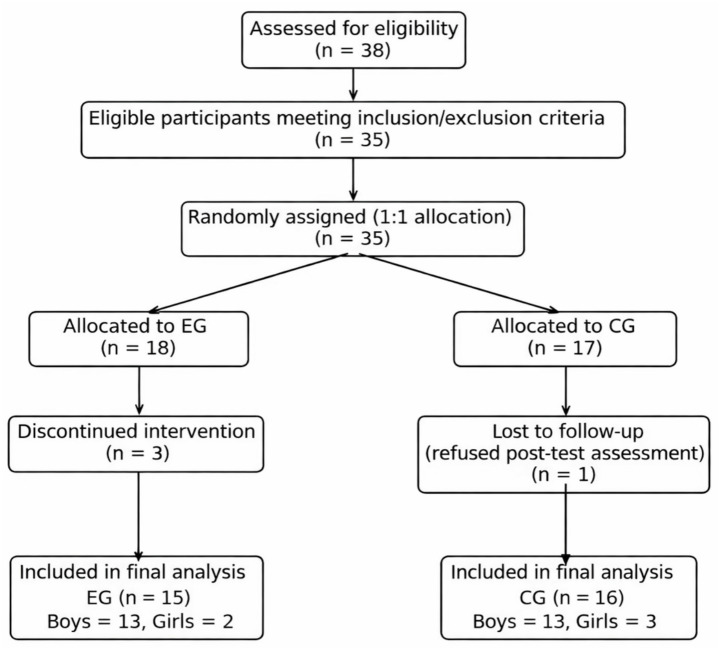
Participant flow diagram.

A total of 38 children were screened, and 35 eligible participants (aged 6–11 years; 28 boys and 7 girls) were enrolled and randomly assigned to the exercise group (EG, *n* = 18) or control group (CG, *n* = 17). The sample exhibited a gender imbalance, with a higher proportion of boys than girls. Although this reflects the typical epidemiological distribution of ADHD, it may limit the generalizability of the findings. During the intervention, three participants in the EG dropped out (two due to scheduling conflicts and one due to excessive absence, defined as missing more than five sessions). In the CG, one participant was excluded at post-test due to non-completion of the follow-up questionnaire. Consequently, data from 31 participants (EG = 15; CG = 16) were included in the final analysis. Reasons for attrition were documented and are presented in the participant flow diagram (Figure 1).

### 2.3 Randomization and blinding

After baseline assessment, participants were randomly assigned to either the EG or a wait-list control group (CG) in a 1:1 ratio using a simple randomization procedure. A random number table was used to generate the allocation sequence, which was prepared by an independent researcher not involved in recruitment, intervention delivery, or outcome assessment. Allocation concealment was ensured by restricting access to the sequence until assignment was completed. Outcome assessors were blinded to group allocation throughout the study. However, due to the nature of the intervention, participants and instructors could not be blinded. In addition, parents or guardians who completed questionnaire-based outcome measures were not blinded, which may have introduced reporting bias (e.g., expectancy effects or social desirability bias). To mitigate this risk, parents were instructed to base their responses on the child’s typical behavior in daily life rather than perceived intervention effects, and the same respondent completed both pre- and post-intervention assessments. Nevertheless, the influence of non-blinding on subjective outcomes cannot be entirely excluded.

### 2.4 Ethics approval and consent to participate

The study protocol was approved by the Biomedical Research Ethics Committee of Henan University (approval number: HUSOM2024-625). Written informed consent was obtained from the parents or legal guardians of all participants prior to participation. All procedures were conducted in accordance with the Declaration of Helsinki.

### 2.5 Exercise manipulation check

Exercise intensity was monitored using heart rate (HR) as an objective manipulation check throughout the intervention. HR was measured using a Polar M430 heart rate monitor (Polar Electro Oy, Finland). During each session, four participants were randomly selected to wear HR monitors during the main exercise phase, following a rotating sampling approach to improve representativeness while minimizing participant burden. Based on the age-predicted maximum heart rate (HRmax = 220 − age), moderate intensity was defined as 60–75% of HRmax (approximately 125–148 bpm). Coaches adjusted task intensity in real time to maintain HR within this target range.

The monitoring results indicated that (Table 1) participants generally maintained HR within the predefined moderate-intensity range, with a mean HR of 132.78 ± 4.29 bpm across sessions. The high proportion of sessions within the target range suggests good intensity compliance. The majority of recorded sessions (>80%) fell within the predefined moderate-intensity range, further indicating high compliance with the intended exercise intensity. Although HR data were collected from a rotating subsample rather than the full sample, the consistency of HR values across sessions indicates stable intensity control. Although full-sample monitoring was not feasible, the rotating sampling approach has been widely used in exercise studies to estimate group-level intensity. Intervention adherence was assessed via attendance records, with an average attendance rate of approximately 94%, indicating high compliance.

**Table 1:** Heart rate responses of four randomly selected participants during mini-basketball intervention sessions.

Session	Participant 1 (bpm)	Participant 2 (bpm)	Participant 3 (bpm)	Participant 4 (bpm)	M (bpm)	SD (bpm)	Exercise intensity zone
1	133.0	128.0	132.0	130.0	130.75	2.22	60–75% HRmax
2	132.0	129.0	130.0	133.0	131.00	1.83	60–75% HRmax
3	130.0	130.0	132.0	130.0	130.50	1.00	60–75% HRmax
4	131.0	130.0	125.0	128.0	128.50	2.65	60–75% HRmax
5	129.0	130.0	138.0	127.0	131.00	4.83	60–75% HRmax
6	131.0	128.0	132.0	130.0	130.25	1.71	60–75% HRmax
…	…	…	…	…	…	…	…
36	135.0	133.0	140.0	138.0	136.50	3.11	60–75% HRmax
Overall	–	–	–	–	132.78	4.29	60–75% HRmax

bpm, beats per minute; M, mean; SD, standard deviation; HR, heart rate; HRmax, maximum heart rate. Detailed HR data collected from four randomly selected participants during each mini-basketball intervention session (36 sessions in total) are provided in Supplementary material 1. A rotating sampling approach was used to ensure representativeness while minimizing participant burden.

### 2.6 Intervention

Participants assigned to the exercise group received a 12-week mini-basketball intervention delivered three times per week, with each session lasting approximately 45 min. The intervention was structured according to children’s developmental characteristics and the EF profile commonly observed in children with ADHD. The program emphasized progressive motor–cognitive engagement rather than competitive performance, with training tasks designed to gradually increase in motor complexity, cognitive demand, and social interaction.

The intervention was implemented in three progressive phases (Table 2). The initial familiarization phase (Weeks 1–2) focused on establishing class routines, basic ball-handling skills, and attentional engagement through simple motor tasks and rule-based activities. The skill acquisition phase (Weeks 3–10) emphasized the learning and practice of fundamental basketball skills (e.g., dribbling, passing, and shooting), along with rule execution, with progressively increasing motor–cognitive demands. The final interaction phase (Weeks 11–12) focused on cooperative games, small-group activities, and simplified gameplay to promote social interaction, emotional regulation, and goal-directed behavior.

**Table 2:** Overview of the mini-basketball intervention program.

Phase	Instructional objectives	Intervention content	Duration	Sessions
Phase 1: Familiarization phase	1. Establish classroom routines and behavioral expectations 2. Improve attention and task completion 3. Reduce novelty-related sensitivity and increase interest in mini-basketball	1. Classroom organization and basic behavioral rules 2. Fundamental ball-handling exercises (e.g., grasping, tapping, tossing and catching) 3. Game-based reaction and attention training (e.g., “Red Light, Green Light”)	2 weeks	6
Phase 2: Skill acquisition phase	1. Enhance learning of complex motor skills 2. Foster task persistence and goal-directed behavior 3. Improve EF	1. Practice of fundamental mini-basketball skills 2. Learning and execution of basic basketball rules (e.g., dribbling, passing, shooting, scoring) 3. Simulated game scenarios emphasizing rule compliance	8 weeks	24
Phase 3: Interaction phase	1. Enhance situational interaction and teamwork 2. Develop social interaction skills and emotional regulation 3. Improve problem-solving and negotiation abilities	1. Cooperative physical games (e.g., group-based chasing and object-transport games) 2. Partner-based cooperative drills and team passing relays 3. Small-sided games with post-game emotional regulation activities	2 weeks	6

Each session consisted of four components (Table 3): (1) class organization and instruction (approximately 3 min), (2) warm-up activities (approximately 5 min), (3) core mini-basketball training and game-based activities (approximately 32 min), and (4) cool-down and relaxation activities (approximately 5 min). Activities were conducted individually, in pairs, or in small groups to promote engagement and adaptability. Exercise intensity was maintained at a moderate level throughout the intervention and was monitored using heart rate assessment, as described in Section 2.5.

**Table 3:** Structure of a single mini-basketball intervention session.

Session component	Content	Format	Objectives	Duration (min)
Opening phase	1. Group assembly and greetings 2. Attendance check and reminder of classroom rules 3. Safety briefing	Whole group	Establish classroom order, clarify behavioral expectations, and reduce anxiety in children with ADHD	3
Warm-up activities	1. Light jogging and dynamic stretching 2. Physical games (e.g., color-based instruction running, reaction training games)	Whole group	Increase heart rate, activate the body, and enhance attention and inhibitory control	5
Mini-basketball training	Phase 1: Ball-handling drills (e.g., bouncing, rolling, tossing and catching) Phase 2: Fundamental basketball skills (dribbling, passing, shooting) Phase 3: Mini-basketball games (e.g., passing relays, cooperative team games)	Individual, pairs, small groups, or whole group	Improve motor skills, promote EF development, and enhance social interaction	32
Cool-down and relaxation	1. Stretching exercises (neck, shoulders, arms) 2. Deep breathing and emotional regulation training 3. Session summary and positive feedback	Whole group	Reduce physical fatigue, facilitate emotional regulation, and consolidate learning outcomes	5

Table 3 illustrates the structure of a representative intervention session (Session 3); the specific content may vary across sessions.

### 2.7 Wait-list control group

A wait-list control design was adopted to ensure that all participants had access to the intervention. Participants in the CG continued their usual daily activities and did not engage in structured exercise programs during the study period. However, this design does not fully control for attention or placebo effects, which should be considered when interpreting the findings. After completion of the study, participants in the CG were offered the same intervention.

### 2.8 Measures

Baseline demographic information, including age, sex, and body mass index (BMI), was collected for all participants. To control for potential confounding factors, habitual physical activity level, ADHD symptom severity, and EF were assessed at baseline and post-intervention using validated questionnaires.

#### 2.8.1 Physical activity rating scale–3 (PARS-3)

Habitual physical activity was assessed using the Physical Activity Rating Scale–3 (PARS-3) (Liang, 1994), which evaluates activity intensity, duration, and frequency over the previous week. A total physical activity score is calculated as intensity × duration × frequency (range: 0–100), with higher scores indicating higher activity levels. Scores ≥ 43 indicate high habitual physical activity. The PARS-3 has demonstrated good reliability and validity in Chinese children and adolescents (Crocker et al., 1997; Li et al., 2015).

#### 2.8.2 Conners’ parent symptom questionnaire—hyperactivity index (PSQ-HI)

ADHD symptom severity was assessed using the Hyperactivity Index of the Conners’ Parent Symptom Questionnaire (PSQ-HI) (Goyette et al., 1978). Parents rated their child’s behaviors on a 4-point Likert scale (0 = not at all to 3 = very much), with higher scores indicating greater symptom severity. The PSQ has demonstrated good internal consistency in Chinese pediatric samples (Su et al., 2001).

#### 2.8.3 Childhood executive functioning inventory (CHEXI)

Executive function can be assessed using neurophysiological methods (e.g., EEG, fMRI), performance-based cognitive tasks (e.g., Stroop, Flanker, N-back), and rating scales. While objective measures provide precise assessments under controlled conditions, they are often resource-intensive and may have limited ecological validity, particularly in pediatric populations with attention difficulties. Therefore, in the present study, the Chinese version of the Childhood Executive Functioning Inventory (CHEXI) (Thorell and Nyberg, 2008) was selected due to its feasibility and its ability to capture children’s EF in everyday contexts. The CHEXI is a caregiver-reported questionnaire consisting of 24 items rated on a 5-point Likert scale (1 = definitely not true to 5 = definitely true). It comprises four subcomponents: working memory, planning, regulation, and inhibition. In accordance with its theoretical structure, working memory and planning reflect the working memory domain, whereas regulation and inhibition represent inhibitory control. All items are reverse scored, with higher scores indicating greater executive dysfunction.

The CHEXI has demonstrated satisfactory psychometric properties. Previous studies have reported good internal consistency (Wei et al., 2018), with Cronbach’s *α* coefficients ranging from 0.71 to 0.89, and acceptable test–retest reliability (0.60–0.69). In addition, CHEXI scores have shown significant correlations with related behavioral constructs, supporting its external validity. The instrument has also been validated across different cultural contexts, with evidence supporting its construct validity. However, as a parent-reported measure, the CHEXI may be subject to reporting bias (e.g., expectancy effects and social desirability), particularly under non-blinded conditions, which may influence the accuracy of the outcomes. This limitation should be considered when interpreting the findings.

### 2.9 Statistical analyses

Statistical analyses were conducted using SPSS version 29.0. Continuous variables are presented as mean ± standard deviation (M ± SD). Baseline differences were assessed using independent-samples *t-*tests or chi-square tests. Within-group changes were analyzed using paired-samples *t-*tests. Between-group differences were examined using analysis of covariance (ANCOVA), with baseline values included as covariates to control for initial group differences. This approach was adopted due to observed baseline imbalances in certain EF outcomes. Although ANCOVA reduces potential confounding, residual bias due to baseline imbalance cannot be completely excluded, particularly given the small sample size. Prior to ANCOVA, assumptions including normality, homogeneity of variances, and homogeneity of regression slopes were tested and satisfied. No significant interaction between group and baseline values was observed, supporting the validity of the ANCOVA model. Effect sizes were calculated using partial eta squared (partial η^2^), with values of 0.01, 0.06, and 0.14 indicating small, medium, and large effects, respectively.

Due to the small sample size and minimal missing data, a per-protocol (PP) analysis was conducted. Intention-to-treat (ITT) analysis was not performed, which may lead to overestimation of intervention effects. Given the exploratory nature of this study, PP analysis was considered appropriate; however, the absence of ITT analysis may limit the robustness of the findings. Future studies are encouraged to incorporate ITT approaches. Sensitivity analyses were not conducted due to the limited sample size, which may further affect the stability of the results. All statistical tests were two-tailed with significance set at *p* < 0.05.

## 3 Results

### 3.1 Baseline characteristics of the study participants

Baseline demographic characteristics and outcome measures for the EG and CG are presented in Table 4. No significant between-group differences were observed at baseline for age, sex distribution, BMI, habitual physical activity level (PARS-3), or ADHD symptom severity (all *p* > 0.05), indicating comparability between groups prior to the intervention. With respect to EF outcomes, baseline scores for inhibitory control and total EF were significantly lower in the EG than in the CG (*p* < 0.05), whereas no significant baseline difference was observed for working memory (*p* > 0.05). Accordingly, baseline EF scores were included as covariates in subsequent analyses to adjust for initial group differences. Although ANCOVA was used to statistically control for baseline differences, the experimental group exhibited significantly lower baseline scores in inhibitory control and total executive function. This initial imbalance may have introduced a potential regression-to-the-mean effect, which could partially inflate the observed intervention effects. Therefore, despite statistical adjustment, the results should be interpreted with caution, as residual confounding effects cannot be entirely excluded, particularly given the relatively small sample size.

**Table 4:** Baseline characteristics of children with ADHD.

Variable	EG (*N* = 15)	CG (*N* = 16)	*t/χ^2^*	*p*
Sex (boy/girl)	13/2	14/2	0.004^#^	0.947
Age (year)	8.07 ± 1.39	7.94 ± 1.44	0.065^#^	0.801
BMI	17.45 ± 0.94	17.33 ± 0.85	0.368^*^	0.716
PARS-3	23.07 ± 5.23	22.88 ± 5.89	0.009^#^	0.925
HI	2.19 ± 0.25	2.19 ± 0.25	0.006^#^	0.938
IC	40.20 ± 1.97	42.38 ± 2.47	−2.696^*^	0.012
WM	40.07 ± 1.58	40.88 ± 1.86	−1.301^*^	0.204
EF	80.27 ± 2.89	83.25 ± 3.80	−2.446^*^	0.021

EG: experimental group; CG: control group; N: number; Mean: mean value; SD: standard deviation; PAR-3: Physical Activity Rating Scale–3 score; HI: hyperactivity index; IC: inhibitory control; WM: working memory; EF: executive function; “*” indicates independent-samples *t*-test; “#” indicates χ^2^ (chi-square) test. BMI: body mass index, calculated as weight (kg) divided by height squared (m^2^). According to the Expert Consensus on Obesity Prevention and Control among Chinese Children and Adolescents, obesity status was determined based on age- and sex-specific BMI cut-off values. Specifically, obesity was defined as BMI > 17.7 for boys and >17.5 for girls aged 6.0–6.5 years; >18.1 for boys and >18.0 for girls aged 6.5–7.0 years; >18.7 for boys and >18.5 for girls aged 7.0–7.5 years; >19.2 for boys and >19.0 for girls aged 7.5–8.0 years; >19.7 for boys and >19.4 for girls aged 8.0–8.5 years; >20.3 for boys and >19.9 for girls aged 8.5–9.0 years; >20.8 for boys and >20.4 for girls aged 9.0–9.5 years; >21.4 for boys and >21.0 for girls aged 9.5–10.0 years; >21.9 for boys and >21.5 for girls aged 10.0–10.5 years; >22.5 for boys and >22.1 for girls aged 10.5–11.0 years; >23.0 for boys and >22.7 for girls aged 11.0–11.5 years; and >23.6 for boys and >23.3 for girls aged 11.5–12.0 years.

### 3.2 Effects of the mini-basketball intervention on EF in children with ADHD

#### 3.2.1 Within-group changes in EF pre- and post-intervention

Within-group analyses revealed significant improvements in EF outcomes in the EG following the 12-week mini-basketball intervention (Table 5). Inhibitory control scores decreased significantly from baseline to post-intervention (t = 6.96, *p* < 0.001). Significant reductions were also observed in working memory scores (*t* = 9.17, *p* < 0.001) and total EF scores (*t* = 13.75, *p* < 0.001). In contrast, no significant pre-to-post changes were observed in the CG for inhibitory control (*p* = 0.188), working memory (*p* = 0.580), or total EF (*p* = 0.270).

**Table 5:** Within-group changes in EF pre- and post-intervention.

Outcome	EG (N = 15)		*t*	*p*	CG (N = 16)		*t*	*p*
	Pre	Post			Pre	Post		
IC	40.20 ± 1.97	39.13 ± 1.89	6.959	< 0.001	42.38 ± 2.47	42.19 ± 2.59	1.379	0.188
WM	40.07 ± 1.58	38.07 ± 1.39	9.165	< 0.001	40.88 ± 1.86	40.69 ± 1.70	0.565	0.580
EF	80.27 ± 2.89	77.20 ± 2.70	13.748	< 0.001	83.25 ± 3.80	82.88 ± 3.91	1.145	0.270

EG: experimental group; CG: control group; Pre: pre-intervention; Post: post-intervention; N: number; M: mean; SD: standard deviation; HI: hyperactivity index; IC: inhibitory control; WM: working memory; EF: executive function.

#### 3.2.2 Between-group differences in EF post-intervention

ANCOVA was conducted to compare post-intervention EF outcomes between groups, with baseline scores entered as covariates. All assumptions for ANCOVA, including normality, homogeneity of variance, linearity, and homogeneity of regression slopes, were met. No significant interaction between group and baseline values was observed, indicating that the assumption of homogeneity of regression slopes was satisfied. Significant between-group differences were observed across all EF domains at post-intervention (Table 6). Compared with the CG, the EG demonstrated significantly lower inhibitory control scores (*F* = 15.15, *p* < 0.001, partial *η*^2^ = 0.35). Significant group effects were also found for working memory (*F* = 30.55, *p* < 0.001, partial *η*^2^ = 0.52) and total EF (*F* = 40.68, *p* < 0.001, partial *η*^2^ = 0.59), indicating large intervention effects favoring the exercise group.

**Table 6:** Between-group differences in EF post-intervention.

Outcome	EG (N = 15)		CG (N = 16)		*F*	*p*	Partial *η*^2^
	Pre	Post	Pre	Post			
IC	40.20 ± 1.97	39.13 ± 1.89	42.38 ± 2.47	42.19 ± 2.59	15.15	<0.001	0.35
WM	40.07 ± 1.58	38.07 ± 1.39	40.88 ± 1.86	40.69 ± 1.70	30.55	<0.001	0.52
EF	80.27 ± 2.89	77.20 ± 2.70	83.25 ± 3.80	82.88 ± 3.91	40.68	<0.001	0.59

EG: experimental group; CG: control group; Pre: pre-intervention; Post: post-intervention; N: number; M: mean; SD: standard deviation; HI: hyperactivity index; IC: inhibitory control; WM: working memory; EF: executive function.

## 4 Discussion

The present study investigated the effects of a 12-week mini-basketball intervention on EF in children with ADHD. The results showed that participants in the intervention group exhibited significant improvements in inhibitory control, working memory, and overall EF, indicating that the program was effective in promoting EF development. These findings are consistent with previous research and recent meta-analytic evidence indicating that structured physical activity generally produces moderate improvements in executive function in children with ADHD (Cerrillo-Urbina et al., 2015; Liu et al., 2025; Pan et al., 2025), with evidence suggesting that cognitively engaging or cognitively combined physical activities may yield greater benefits, highlighting the important role of task complexity and cognitive engagement in shaping intervention effects (Fang et al., 2024). In addition, preliminary evidence from earlier studies suggests that basketball-related training may improve specific cognitive functions. For example, Eskandarnejad et al. (2015) reported improvements in working memory following short-term basketball training in children with ADHD. However, such studies are generally limited by small sample sizes and relatively weak methodological designs. Notably, the magnitude of improvement observed in the present study appears greater than that reported in many previous exercise-based interventions, which have typically demonstrated moderate effects on inhibitory control and working memory. One possible explanation is that the mini-basketball program incorporated a higher level of cognitive engagement, integrating physical activity with rule-based decision-making, response inhibition, and continuous updating of action plans.

Several mechanisms may potentially account for the observed improvements. First, moderate-intensity physical activity likely provided a fundamental physiological basis for EF enhancement. Repeated engagement in dynamic motor activities (e.g., running, jumping, and rapid directional changes) may promote cerebral blood flow and facilitate neurochemical processes associated with executive control, including catecholaminergic modulation (Hillman et al., 2008; Guzmán-Muñoz et al., 2025; Vaynman and Gomez-Pinilla, 2005). Beyond these general physiological effects, the cognitively demanding nature of the intervention may have played a key role. Mini-basketball requires continuous monitoring of environmental information, adherence to rules, inhibition of inappropriate responses, and flexible adjustment to rapidly changing situations, which may impose sustained demands on core EF components, particularly inhibitory control and working memory (Best, 2010; Diamond and Lee, 2011). Importantly, these demands were embedded within a structured and progressive training framework, which may have facilitated repeated engagement and reinforcement of executive processes over time (Tomporowski et al., 2015; Pesce, 2012). In addition, social interaction embedded within the intervention may have provided supplementary benefits to EF development. Cooperative and team-based activities require children to follow rules, regulate behavior, and coordinate actions with peers, which are closely related to self-regulation and executive control (Christiansen et al., 2019; Wang et al., 2025).

However, these mechanisms were not directly measured in the present study and should therefore be interpreted with caution.

## 5 Conclusion and limitations

This study provides evidence that a 12-week mini-basketball program significantly improves inhibitory control, working memory, and overall EF in children with ADHD. These findings support the potential of developmentally appropriate, structured, and cognitively engaging mini-basketball interventions as a feasible non-pharmacological strategy to enhance EF in this population.

Despite these strengths, several limitations should be acknowledged. First, baseline differences in inhibitory control and overall EF were observed between groups. Although randomization was employed, such imbalances are not uncommon in studies with relatively small sample sizes. To address this issue, baseline values were included as covariates in the ANCOVA, which is a commonly recommended approach for adjusting initial group differences. However, statistical adjustment cannot fully eliminate potential bias, and residual confounding may still have influenced the estimated intervention effects. Therefore, the results should be interpreted with caution, particularly regarding the magnitude of the observed effects. Second, a per-protocol (PP) analysis was conducted due to the small sample size and the limited proportion of missing data, whereas intention-to-treat (ITT) analysis was not performed. The absence of ITT analysis may increase the risk of overestimating intervention effects, as participants who discontinued the study were not included in the final analysis. In addition, sensitivity analyses were not conducted to assess the robustness of the findings under different analytical assumptions. Future studies should incorporate both ITT and sensitivity analyses to enhance methodological rigor. In addition, the relatively small sample size, gender imbalance (with a predominance of boys), and single-site recruitment may limit the generalizability of the findings. Although this distribution reflects the typical epidemiological characteristics of ADHD, caution is warranted when extending the results to more diverse populations and broader clinical contexts. Finally, although the intervention was associated with improvements in EF, the underlying mechanisms were not directly assessed in the present study. In addition, EF outcomes were primarily based on a parent-reported questionnaire, which, despite its ecological validity, may be subject to reporting bias (e.g., expectancy effects and social desirability), particularly in the absence of full blinding. These factors limit the ability to draw definitive conclusions regarding the neurocognitive or physiological processes underlying the observed effects.

Future research should incorporate multi-method approaches, including performance-based cognitive tasks and neurophysiological measures (e.g., EEG or fMRI), to provide a more comprehensive and objective evaluation and to clarify the underlying mechanisms.

## Data Availability

The datasets presented in this study can be found in online repositories. The names of the repository/repositories and accession number(s) can be found in the article/Supplementary material.

## Glossary

ADHD: Attention-deficit/hyperactivity disorder
BMI: Body mass index
bpm: Beats per minute
CG: Control group
CHEXI: Childhood Executive Functioning Inventory
DSM-5: Diagnostic and Statistical Manual of Mental Disorders, Fifth Edition
EF: Executive function
EG: Exercise group
Heart rate: HR
HI: Hyperactivity Index
HRmax: Maximum heart rate
IC: Inhibitory control
IQ: Intelligence quotient
M: Mean
N: Number
PARS-3: Physical Activity Rating Scale
partial η^2^: Partial eta squared
PSQ-HI: Conners’ Parent Symptom Questionnaire—Hyperactivity Index
RCT: Randomized controlled trial
SD: Standard deviation
WM: Working memory
